# Translation, Cross-Cultural Adaptation, and Validation of the Croatian Version of the Athlete Psychological Strain Questionnaire (APSQ)

**DOI:** 10.3390/sports12080228

**Published:** 2024-08-22

**Authors:** Katarina Sore, Frane Franic, Luka Androja, Ivana Batarelo Kokic, Darko Marčinko, Stipe Drmic, Zdravko Valentin Markser, Tomislav Franic

**Affiliations:** 1 School of Medicine, University of Split, 21000 Split, Croatiatomislav.franic@mefst.hr (T.F.); 2 School of Medicine, University of Zagreb, 10000 Zagreb, Croatia; 3 Department of Sports Management, Aspira University of Applied Sciences, 21000 Split, Croatia; 4 Faculty of Humanities and Social Sciences, University of Split, 21000 Split, Croatia; batarelo@ffst.hr; 5 Department of Psychiatry, University Hospital Zagreb, 10000 Zagreb, Croatia; 6 Department of Psychiatry, Dubrava University Hospital, 10000 Zagreb, Croatia; sdrmic@kbd.hr; 7 Department of Health Studies, University of Split, 21000 Split, Croatia; 8 University of Applied Health Sciences, 10000 Zagreb, Croatia; 9 German Society for Sport Psychiatry and Psychotherapy, 50668 Cologne, Germany

**Keywords:** athletes, mental health, assessment, validation, surveys, questionnaires

## Abstract

The aim of this study is to translate, cross-culturally adapt, and validate the Croatian Athlete Psychological Strain Questionnaire (APSQ-Cro) as part of the Sport Mental Health Assessment Tool 1 (SMHAT-1) validation. We assessed the reliability and applicability of the APSQ-Cro among Croatian athletes. The international sports community is increasingly focused on mental health issues in athletes, highlighting the need for early detection tools like the Athlete Psychological Strain Questionnaire (APSQ) and SMHAT-1. We included 869 Croatian competing athletes across 54 sports who received a link to access the WEB-based questionnaire. The Croatian Olympic Board helped in distributing the questionnaires, aiming to reach as many and as diverse a group of registered competing athletes in Croatia as possible. Results showed a Cronbach’s alpha of 0.75 for the entire questionnaire, indicating acceptable reliability. An exploratory strategy of factor analysis was used to determine the underlying structure of the APSQ-Cro. For this purpose, the Kaiser–Meyer–Olkin (KMO) test and Bartlett’s test for sphericity were performed to ensure the suitability of the data. The KMO test ensured sampling adequacy, with a measure of 0.77 indicating suitability for factor analysis, while Bartlett’s test confirmed significant correlations among variables (χ^2^ = 2779.155, df = 45, *p* < 0.001), validating the dataset’s appropriateness for data reduction techniques. The factor analysis, together with the Cattell scree test and varimax rotation, resulted in a two-factor structure for the APSQ-Cro. Factor 1 included items related to internal psychological struggles, while Factor 2 included items related to external pressures from the athletic environment. These two factors explained 53% of the variability, with Cronbach’s alphas of 0.75 and 0.88 for the respective factors. The APSQ-Cro is a valid and reliable tool for assessing distress in Croatian athletes. Croatian athletes’ sporting experience will be improved with the broad adoption of the APSQ-Cro, which can help detect early signs of psychological distress and subsequently improve mental health outcomes.

## 1. Introduction

There is increasing importance placed on athletes’ daily mental health, with clinical research on the subject being conducted globally, including in Europe [1,2], the USA [3], and China [4]. To demonstrate the increasing focus on athletes’ mental health, we conducted a brief ad hoc research study in PubMed covering the period from 2000 to 2024. Our goal was to show the significant rise in the number of related articles. Initially, there were 250 articles published between 2000 and 2005. This number doubled approximately every five years: 483 articles from 2005 to 2010, 963 from 2010 to 2015, and 2064 from 2015 to 2020. Despite the last interval being shorter (2020 to April 2024), there were still 1707 articles, highlighting a continued increase and growing academic interest in this area.

A study from Sweden provides an overview of top athletes and high-performance coaches with psychiatric disorders in two publicly funded clinics. The results showed that anxiety disorders were most common in top athletes (69%). Stress-related disorders were found in 25% of elite athletes. Affective disorders were found in 51% of elite athletes. Eating disorders were widespread among elite athletes (26%), particularly among women (37%). Almost three in four high-performance coaches suffered from stress and adjustment disorders, compared to one in four elite athletes. Eating disorders were found in about one in four athletes, and about one sixth of high-performance coaches suffered from a substance use disorder [5].

Recent studies have shown that there are significant mental health problems among top athletes. A Swiss study of 1003 athletes found that 17% suffered from depression, 10% from anxiety, 22% from eating disorders, and 18% from sleep disorders, with women more likely to suffer from mental disorders compared to men. Injured athletes were particularly affected by depressive symptoms. The study highlights the importance of need satisfaction, frustration, and perceived organizational support in relation to athletes’ well-being and mental health [6]. Research on ultra-endurance athletes shows a higher prevalence of psychiatric disorders, including depression and anxiety, related to intense training and high motivation. This group faces unique psychological challenges that require tailored mental health support [7]. A Norwegian study of 378 elite athletes found that 74.1% of athletes had ‘at risk’ scores for mental health disorders, with sleep problems and body dysmorphic disorder being the most common issues. The results point to the need for longitudinal studies and warn against relying solely on self-reporting for diagnosis [8]. An international think tank of the International Society of Sport Psychology has produced a consensus statement advocating a comprehensive approach to the mental health of athletes and emphasizing the need for clear definitions, increased organizational support, and the appointment of mental health officers in sports organizations [9]. Overall, while awareness of mental health issues in sport has increased, significant gaps in service delivery and treatment remain, highlighting the urgent need for improved interventions and resources to effectively support athletes’ mental health [10].

It is well established that while sport can offer numerous benefits, it also carries significant mental health risks, particularly for those who train at a competitive level. Addressing these issues through a supportive environment, mental health resources and awareness is vital to athletes’ wellbeing. For example, athletes, especially at the elite level, are often under tremendous pressure to perform, which can lead to chronic anxiety and depression. The stress associated with competition can set in motion a cycle of mental health problems, as demonstrated by the experiences of elite athletes who have started to speak openly about their problems with anxiety and depression despite their successes [11]. Furthermore, research has shown that athletes often suffer from sleep disorders, which can exacerbate mental health problems. Poor sleep quality has been linked to increased levels of depression and anxiety in athletes, creating a vicious cycle that affects both performance and overall well-being [12]. The sporting environment can sometimes be toxic, with bullying and abuse from coaches, teammates, and fans contributing to mental health problems. This can manifest in low self-esteem, anxiety, and even suicidal thoughts, especially in young athletes who are particularly vulnerable to such negative influences [11,13]. The pressure to maintain a certain body image and performance level can lead to eating disorders and sports addiction. Athletes can develop an unhealthy relationship with food and exercise, which can lead to psychological problems such as anxiety and depression. Studies indicate that athletes, especially those in weight-sensitive sports, are at higher risk of these disorders [11,13]. The phenomenon of burnout is widespread among athletes and is characterized by emotional exhaustion, a drop in performance, and a feeling of detachment. This condition is particularly common in youth sports, where overtraining and early specialization can lead to significant psychological problems [13,14]. Recent discussions in the medical community have pointed to a mental health crisis in athletes, particularly adolescents. Factors contributing to this crisis include the increasing professionalization of sport, a lack of mental health resources, and societal pressures [14].

Regular exercise is associated with enhanced mental health, benefiting individuals with mild to moderate depression and anxiety by promoting routine physical activity for mental well-being [15]. Dinas et al. explored exercise’s role in stimulating endorphins, natural mood enhancers, and pain relievers; their findings support the “endorphin theory” of exercise as an antidepressant-like therapeutic [16]. Singh et al.’s systematic review assessed physical activity’s efficacy in reducing depression and anxiety symptoms, concluding that various adult populations, including those with mental or chronic illnesses, benefit significantly from physical activity [17]. There is increasing interest in the negative impacts of sports on athletes too, emphasizing early detection and intervention. Research indicates that athletes are as prone to mental disorders as the general population, with specific physical stresses impacting their mental health [18]. Reardon and Factor stress the need for more research to understand the prevalence, risk factors, and effects of mental health issues in athletes. They point out the infancy of this field, hindered by scant research and a lack of tailored diagnostic and treatment strategies. The review emphasizes the importance of mental health care in maintaining athletes’ careers, noting complications from stigma and limited research [19]. The International Olympic Committee (IOC) recognizes that mental health issues are common among elite athletes, affecting both their performance and physical health, yet no standardized guidelines exist for diagnosing and treating these disorders. The IOC calls for a more consistent, evidence-based approach to mental health management in elite sports [20]. International sports organizations and experts underscore the necessity of enhancing mental health care for athletes [3,21,22,23]. Furthermore, the connection between elite athletes’ mental health and their performance and injury risk underscores the need for reliable instruments for early detection of stress and mental health symptoms [24]. The need for early detection of mental health issues in athletes has led to the development of tools like the Athlete Psychological Strain Questionnaire (APSQ), which can function independently or as part of a broader assessment. The International Olympic Committee (IOC) has introduced the Sport Mental Health Assessment Tool 1 (SMHAT-1), incorporating the APSQ as the initial triage tool. We decided to translate and validate the entire SMHAT-1 with the APSQ as the first step since it serves as the triage instrument [25].

The APSQ is a questionnaire developed by Australian researchers in 2018 [24]. The APSQ items were designed to assess problems with teamwork, poor impulse control and frustration tolerance, concerns about training stress and athletic performance, and adjustment to life after professional sport [24]. The 10 items of the APSQ self-report questionnaire are The 10 items of the APSQ self-report questionnaire are: It was difficult to be around teammates, I found it difficult to do what I needed to do, I was less motivated, I was irritable, angry, or aggressive, I could not stop worrying about injury or my performance, I found training more stressful, I found it hard to cope with selection pressures, I worried about life after sport, I needed alcohol or other substances to relax, and It was difficult to be around teammates. The Athlete Psychological Strain Questionnaire (APSQ) measures the psychological distress of athletes in three dimensions—performance, external coping, and self-regulation—in the past 30 days on a 5-point scale from “never” to “always” (1 = “never”; 5 = “always”). The performance dimension includes items such as APSQ5, which reflects concerns about injury and performance. External coping assesses how athletes deal with external pressures, represented by APSQ6 and APSQ7, which focus on stress from training and selection pressure. Self-regulation covers internal psychological struggles and emotional regulation, including items APSQ1, APSQ2, APSQ3, APSQ4, APSQ8, APSQ9, and APSQ10. These items cover difficulties with teammates, motivation, aggression, worries about life after sport, and dependence on recreational substances. Taken together, these items provide a comprehensive assessment of an athlete’s psychological distress. Scores range from 10 to 50, with higher scores indicating greater psychological distress [24]. Validity and reliability have been confirmed, and it is available in Japanese [26], Arabic [27], Turkish [28], and Chinese [29], showing satisfactory reliability but differences in factorial analysis and differences between the number of sports and the number of athletes included in APSQ development and validation (Table 1). Furthermore, those differences include gender inclusion (some surveys included only male athletes). If we compare the number of participants with the country population at the time of the survey, we can notice differences there, too (Table 1).

Currently, we are not aware of validated mental health assessment tools for athletes available in Croatian. The aim of this study is to translate, validate, and test the reliability and internal consistency of the APSQ among Croatian male and female athletes, leading to the development of the Croatian version of the APSQ (APSQ-Cro). We hypothesize that the APSQ-Cro has satisfactory reliability and validity and is therefore a useful instrument for the early detection of mental health problems in Croatian athletes. The aim is to improve mental health support for athletes through early intervention and targeted resources.

## 2. Materials and Methods

### 2.1. Study Design

This cross-sectional study followed the Strengthening the Reporting of Observational Studies in Epidemiology (STROBE) guidelines [30].

#### 2.1.1. Translation and Intercultural Adaptation

The original SMHAT-1 questionnaire was in English and required translation into Croatian. A bilingual researcher performed the forward translation, and a Croatian professor reviewed it, suggesting two minor adjustments to enhance clarity. These corrections were incorporated into the draft APSQ. Subsequently, a court-certified English interpreter, fluent in Croatian, conducted the back-translation. The bilingual research team (native Croatian speakers fluent in English) compared the original APSQ with its back-translation to identify any linguistic or cultural discrepancies, finding none. This process led to the creation of the Croatian draft APSQ (Draft APSQ-Cro). All translators recognized the critical nature of their work, prioritizing cultural relevance and accuracy to ensure the document’s appropriateness for the target population. The research team also compiled sociodemographic questions covering gender, age, education, family, and social dynamics, along with sports participation. These were added to the APSQ questionnaire to enhance understanding of the athletes’ mental health, which correlates with these sociodemographic factors. We added 28 sociodemographic questions to the translated APSQ and SMHAT-1, forming the APSQ-Cro draft, which was then hosted on Google Forms.

#### 2.1.2. Comments from the Expert Group

From 22 May to 10 June 2023, a twelve-member expert panel—comprising four psychiatrists, three sports psychologists, and five language professors—reviewed the draft SMHAT-1 and APSQ-Cro, including the sociodemographic questions. They accessed the questionnaire via emailed links and barcodes, completing it at their convenience.

After accessing the questionnaire on Google Forms, the expert group members consented to participate by checking a box, having been informed about the study’s purpose and data handling. The questions were uniformly presented online. Experts provided feedback via email on four aspects:

1. user-friendliness of the questionnaire;
2. appropriate length of the questionnaire;
3. clarity of the questionnaire and;
4. an open field for additional comments.

Comments from the expert review aided the cross-cultural adaptation and identified unclear elements. While there were no issues with the APSQ itself, feedback on the sociodemographic questions suggested some were unnecessary and overly complex. Consequently, we reduced the number of questions from 28 to 25, simplified others, and decreased the number of answer choices.

#### 2.1.3. The Pilot Group

Conducted in July 2023, our pilot study included 20 athletes from Split and its vicinity who met the following inclusion criteria: 16 to 65 years old, had competed in the last 12 months, and trained more than 10 h a week. Athletes in the pilot study received the questionnaire via email, identical to the expert group, and provided feedback on the same four questions. Feedback highlighted issues only with the sociodemographic questions, describing some as overly lengthy with excessive answer options. Consequently, we revised four questions, changed another four, and reduced the answer choices for four. Following these revisions, we resubmitted to the Medical University of Split’s Ethics Committee to amend our existing approval and received the updated approval in September 2023.

#### 2.1.4. The Validation Sample and Participants

To minimize selection bias, the questionnaire was emailed to athletes through the Croatian Olympic Board (COB), sports organizations in each county, and associations in every mayoral town, ensuring broad distribution across diverse sports. The email containing access details and a barcode was sent twice to ensure broader reach across the databases. The COB and associated sports bodies were instructed to forward this email to all athletes in their databases. At the beginning of the questionnaire on Google Forms, participants received detailed information about its purpose and data handling, and consented to the study by checking an appropriate box. From September to December 2023, we validated the questionnaire with 869 athletes who met the criteria active in 54 sports.

The demographic data show that the highest percentage of participants were involved in swimming (21.05%), closely followed by soccer (19.90%), athletics (11.27%), and tennis (9.89%). In terms of gender distribution, 60.87% of participants were men and 38.55% were women. In terms of education, a clear majority, 62.02%, had completed secondary school. Marital status showed that 53.04% of the participants were not in a relationship, while 39.81% were in a relationship. In addition, 28.20% of the athletes stated that they had suffered an injury in the past year, while 71.80% had not. On average, the participants had taken part in 14.27 competitions in the last 12 months.

In terms of the representativeness of the sample, it covered a broad spectrum of athletes from 54 different sports in Croatia, with significant gender representation and a wide age range. The recruitment strategy consisted of sending the questionnaire to athletes via email through the Croatian Olympic Committee (COB), sports organizations in each county, and federations in each major city to ensure wide distribution. This method improved the generalizability of the results, as athletes from different sports, performance levels, and regions of Croatia were included. Representativeness was enhanced by capturing different levels of education, marital status, and sports, reflecting the diversity within the sporting population in Croatia.

Incomplete questionnaires missing more than 20% of the APSQ-Cro responses were excluded, resulting in the removal of 25 questionnaires. This ensured the reliability and validity of the results by avoiding bias in factor structure and reliability analyses.

#### 2.1.5. Statistical Analysis

Descriptive statistics parameters were calculated for two reasons, to describe the demographic characteristics of the athletes and to create descriptive statistics for the APSQ items. With a population of 15,245 athletes registered with the Croatian Olympic Committee, the required sample size is approximately 372 to achieve a 95% confidence level with a 5% margin of error. A total of 869 Croatian athletes participated in the study, which exceeds the calculated sample size. This larger sample size increases the reliability and validity of the results and allows for more robust statistical analyses.

Exploratory factorial analysis (EFA) was chosen for this study to determine the underlying structure of the APSQ-Cro for several reasons. First, there is no validated instrument for assessing athletes’ mental health in Croatian, which makes EFA an appropriate method to explore and uncover the latent constructs within the APSQ-Cro without preconceived assumptions. The EFA approach allows for data-driven discovery of factor structure, which is crucial in a new cultural context where linguistic and cultural nuances can influence item responses differently. In addition, the appropriate sample size (869 athletes) supports the reliability of the EFA results. The Kaiser–Meyer-Olkin (KMO) measure of sampling adequacy was 0.77, indicating the suitability of the data set for factor analysis. Bartlett’s test for sphericity (χ^2^ = 2779.155, df = 45, *p* < 0.001) confirmed the significant correlations between the variables, justifying the use of data reduction techniques. In addition, EFA represents a first step in the validation process and allows the identification of a factor structure that can subsequently be tested and confirmed in future studies using confirmatory factor analysis (CFA). This method ensures that the APSQ-Cro is based on the actual responses of Croatian athletes, making it a reliable and culturally relevant tool for assessing psychological distress.

By using the Cattell scree test and varimax rotation, the study ensured that the factors determined were not only statistically significant but also practically meaningful and interpretable, which is crucial for an effective assessment of the mental stress of athletes. Factor structures have been presented together with the variance accounted for each factor (Expl. Var.) and the proportion of variability accounted for each factor (Prp. Totl.). As a measure of reliability for the whole scale and each subscale, Cronbach’s α (Cα) was calculated. All calculations have been undertaken using the statistical package Statistica 14.1.0.8 (Cloud Software Group Inc. Split, Hrvatska).

## 3. Results

This research included athletes who in major part were involved in swimming (20.21%), soccer (19.7%), athletics (11.2%), and tennis (9.8%). Descriptive statistics and sensitivity analyses confirmed that excluding these questionnaires did not significantly impact the study’s findings (Table 2).

Descriptive statistics for each item were calculated (Table 3) [31]. For APSQ1, the median was 1.00, which suggests a skew towards lower values or less variation among responses. APSQ6 showed a median of 3.00, indicating the responses lean towards a higher value compared to others. A higher standard deviation (as in APSQ7) indicates more varied responses, while a lower value (as in APSQ9) suggests responses are more clustered around the mean [32,33].

### 3.1. Assessing Suitability for Factor Analysis

#### 3.1.1. Kaiser–Meyer–Olkin (KMO) Test

An MSA of 0.77 indicated that the sampling adequacy for the dataset was suitable for factor analysis. High MSA values were found in: APSQ1, APSQ2, APSQ3, APSQ4, APSQ5, APSQ8, APSQ9, and APSQ10, with APSQ 1 particularly well-suited for factor analysis. Low MSA values were evident for APSQ6 and APSQ7, indicating that these two items may not be suitable for reliable factor analysis or suggesting that they belong to another factor.

#### 3.1.2. Bartlett’s Test of Sphericity

The results of Bartlett’s test (χ^2^ = 2779.155, df = 45, *p* < 0.001) suggested that there was a significant correlation between the variables, and that a data reduction technique would be suitable for the dataset.

### 3.2. Exploratory Factor Analysis

APSQ factorization (Table 4) identified two factors among the APSQ items.

Factor 1 includes items that relate to internal psychological struggles, while Factor 2 includes items that relate to external pressures from the sporting environment. Specifically, Factor 1 applied to APSQ1, APSQ2, APSQ3, APSQ4, APSQ5, APSQ8, APSQ9, and APSQ10, while Factor 2 applied to APSQ6, APSQ7. The factor loadings and details of the items can be found in Table 4, which shows the correlation between the individual items and the identified factors and thus helps to clarify which items belong to which factor. The total variance proportion was 53%, with Factor 1 being more dominant at 35% and Factor 2 at 18%.

### 3.3. Validity Test

**Figure 1:**
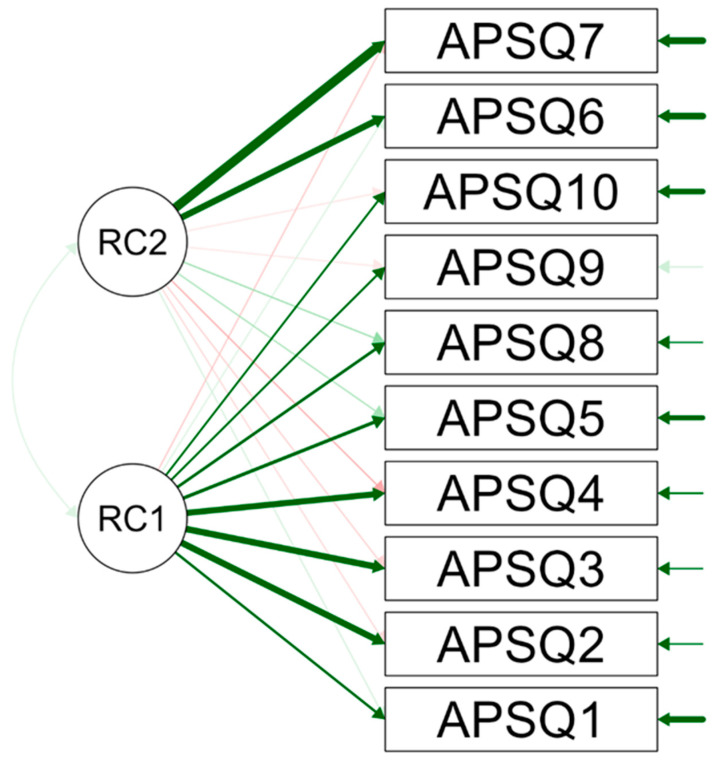
Path diagram for APSQ: Athlete Psychological Strain Questionnaire.

Factor characteristics provide results for factor analysis both before and after rotation, focusing on the characteristics of the two factors identified. The unrotated solution demonstrated eigenvalues for Factor 2 alone, showing a lesser contribution compared to Factor 1 but still significant. The rotated solution (varimax rotation) showed that both factors retain the same sum of squared loadings post-rotation for Factors 1 and 2. The proportions of variance explained remain unchanged for both factors. The rotation did not change the proportions of the variance explained or the cumulative variance, suggesting that the initial factor structure was already clearly defined. Figure 1 depicts a factor-loading diagram from a factor analysis, showcasing the relationships between all individual APSQ items and the two underlying factors, labeled RC1 (Factor 1) and RC2 (Factor 2).

Figure 1 illustrates this using a path diagram showing the relationships between the individual APSQ elements and the two underlying factors. The diagram illustrates that APSQ1–APSQ5, APSQ8, APSQ9, and APSQ10 load more heavily on Factor 1 (RC1), while APSQ6 and APSQ7 load more heavily on Factor 2 (RC2).

### 3.4. Reliability Assessment

Cronbach’s α for the entire scale was 0.75, reflecting acceptable internal consistency with a 95% confidence interval (CI 0.73–0.79). Further statistical analysis for Cronbach’s α, revealed a Cronbach’s α for Factor 1 of 0.81 and for Factor 2 of 0.85. Both represent reliability, including confidence intervals (CI) of 95%. These results indicate that Factor 2 has a slightly better internal consistency and reliability in measuring the underlying construct. This indicates better internal consistency for F2, which correlates with the factorization results.

In terms of frequentist individual item reliability statistics (Table 4), APSQ2 and APSQ3 showed relatively higher item–rest correlations. These items were well aligned with the construct being measured and contributed positively to the scale [34]. APSQ6 and APSQ7 showed lower item–rest correlations. This again correlated with the factorization results [35].

## 4. Discussion

After careful analysis of the primary data, we demonstrated the validity and reliability, and found the factor structure, of the APSQ-Cro, which revealed a two-factor structure. It was identified that eight items are related to Factor 1 (internal psychological struggles) and two items are related to Factor 2 (external pressures). The Croatian, Saudi Arabian, Chinese, Turkish, and Japanese versions of the Athlete Psychological Strain Questionnaire (APSQ) show high internal consistency across all adaptations. Each version was validated for its effectiveness in assessing athletes’ psychological strain. These values indicate good reliability across different language adaptations. With comparatively high Cronbach’s alphas, the APSQ-Cro can be regarded as a helpful assessment tool for athlete-specific psychological stress in Croatian competing athletes.

Additionally, the mean total scores for the 10-item APSQ indicate that athletes in Australia and Croatia experience similar and higher levels of psychological stress compared to athletes in Japan. However, the lower mean score in Japan could be due to cultural differences in perceiving and reporting psychological distress, with Japanese norms potentially leading to underreporting. Studies indicate that athletes from collectivist cultures may underreport psychological distress due to social norms surrounding mental health [36]. Studies also show the role of the training environment in shaping athletes’ mental health. For instance, a supportive coaching style and positive team dynamics have been linked to better mental health outcomes [37]. The Croatian version contained two factors which might be the most intriguing finding, since the factor structure varies across studies. The original, Australian, Chinese, and Turkish versions had three, and the Japanese version showed one [24,26,28,29]. Based on the factor loadings, the grouping of items into two factors might reflect underlying themes related to internal (personal) and external (environmental) pressures experienced by athletes.

The first factor includes items capturing difficulties being around teammates, challenges in performing necessary tasks, decreased motivation, irritability or anger, constant worries about injury or performance, and concerns about life after sports. These items suggest a focus on internal psychological struggles, including emotional regulation and personal anxiety. They may reflect how athletes internally process and respond to the psychological demands of their sports careers. This factor might indicate the personal resilience and internal coping mechanisms athletes employ or struggle with in their sporting environments. The literature suggests that building resilience can mitigate the effects of stressors [38]. The concerns about post-sport life also suggest worries about identity and future, which are deeply personal issues. We propose naming this factor as Intrapersonal Psychological Strain (APSQ1, APSQ2, APSQ3, APSQ4, APSQ5, APSQ8, APSQ9, APSQ10).

The second factor captures stress from training and coping with selection pressures. These items are oriented towards external aspects of an athlete’s career, specifically the pressures and stresses from the sports environment itself, such as the demands of training regimes and the stress of being selected for competitions. This factor might encompass how external factors such as coaching, team selection, and training intensity influence an athlete’s mental health. It addresses the broader context in which an athlete operates rather than their internal coping mechanisms. We propose naming this factor as Environmental Psychological Strain (APSQ6, APSQ7).

The factor structure of stressors for athletes can be divided into personal (affecting internal states and feelings) and external (related to physical environment and career structure). This distinction is crucial for developing appropriate interventions.

Successful intervention strategies, for instance cognitive–behavioral approaches, have been effective in addressing anxiety and depression in athletes [39]. The literature indicates that male and female athletes may experience and express psychological strain differently, necessitating gender-specific interventions [40].

Personal stressors may need psychological and therapeutic approaches to enhance internal resilience, while environmental stressors could be managed through adjustments in training practices, team dynamics, and selection processes.

The noticed factor differences are important and need to be clarified in further research. So far, we can offer some plausible explanations for different factor structures. Sample characteristics can give variations because cultural, demographic, or professional background can influence responses. “Tightness Score” reflects social norms and deviance tolerance, varying widely across countries, potentially contributing to the difference in factors between the APSQ and APSQ-Cro [41]. It should be noted that differences in cultural perceptions of help-seeking between cultures are well known [42]. Studies have also described cross-cultural differences in emotional self-perception [43]. Despite rigorous translation, subtle linguistic and cultural differences can affect question interpretation, potentially altering factor structures. Variations in questionnaire versions or contexts (clinical vs. general population, different sports) may also impact results.

### 4.1. Strengths and Limitations of This Research

The study included 869 athletes from 54 sports across Croatia, enhancing the findings’ generalizability. The authors aimed to cover all competing athletes in clubs/associations, achieving significant gender and sports diversity and representing 0.022% of the target population. The APSQ-Cro showed acceptable reliability with a Cronbach’s alpha of 0.75 and a two-factor structure explaining 53% of the variability, validating its use for assessing psychological strain in Croatian athletes. The translation process ensured cultural relevance and accuracy. However, the large number of clubs limited our control over questionnaire distribution, potentially causing sampling biases. The self-reported nature of the APSQ-Cro might introduce response biases, and non-response bias could affect the results if non-responders differ from responders. Additionally, the proportion of total variance leaves a portion of the variance unexplained, indicating other factors not captured.

### 4.2. Implications for Future Research

Further research is needed to test the APSQ across different cultures, genders, and sports settings to ensure its effectiveness. Longitudinal studies could validate its predictive power and clinical utility by correlating APSQ scores with long-term mental health outcomes and performance in athletes. Based on diverse findings, the APSQ could be refined to better address relevant psychological strains. The development of the APSQ-Cro offers a unique opportunity for future research in Croatian sports, where mental health studies among competitive athletes are limited. This study is part of a larger project developing the Croatian version of SMHAT-1, aiming to explore the APSQ as a standalone tool and its correlation with SMHAT-1 subscales.

Future research should focus on the following: Conducting a confirmatory factor analysis (CFA) to validate the two-factor structure identified in the exploratory factor analysis; examining convergent validity by assessing the relationships between the APSQ-Cro and other established measures of psychological distress and mental health; assessing discriminant validity by comparing the APSQ-Cro with measures of unrelated constructs; and conducting an invariance analysis to examine whether the APSQ-Cro varies across different groups, such as gender and age categories. Gender and age categories should be considered. Longitudinal studies could also contribute to mental health maintenance by tracking changes in psychological distress over time, providing insights into the predictive validity of the APSQ-Cro and its utility in monitoring athletes’ mental health over time. Cross-cultural comparisons will help to understand how the APSQ-Cro works in different cultural contexts in order to refine the instrument for broader international use and improve its generalizability.

These additional analyses will further strengthen the reliability and validity of the APSQ-Cro and ensure its effectiveness in assessing psychological distress among Croatian athletes.

### 4.3. Novelty and Importance

According to our knowledge, this is the first tool that explores mental health in athletes in Croatian.

## 5. Conclusions

We have developed the APSQ-Cro, a tool designed for assessing the mental health status of Croatian competing athletes. The APSQ-Cro showed a satisfactory level of validity, supported by appropriate statistical analyses, in a representative sample of 869 Croatian competing athletes.

We hope that the APSQ-Cro will be accepted in the Croatian sports sector, where it could help in the early detection of psychological stress. Early detection of psychological stress ensures timely help and can improve the mental health of athletes, prevent injuries, and improve athletic results.

## Figures and Tables

**Table 1:** Sport diversity and gender representation in APSQ surveys.

Country	Survey Year	Number of Participants	Total Population	Percentage of Total Population	Sports Count	Male Athletes	Female Athletes	Non-Disclosed
Australia	2018	1007	25.2 M	0.0039%	3	1007	0	0
Japan	2021	612	125.1 M	0.00048%	1	612	0	0
Saudi Arabia	2021	98	35.95 M	0.00027%	N/A	76	31	0
Turkey	2021	565	84.6 M	0.00066%	21	318	247	0
China	2022	406	1.43 B	0.0000028%	N/A	N/A	N/A	N/A

N/A: Data not available. Total population figures are based on estimates from the respective survey years. Percentage of total population is calculated as (Number of Participants/Total Population) × 100.

**Table 2:** Demographic characteristics of the athletes.

Category	Subcategory	Statistics
Gender	Male	529 (60.87%)
	Female	335 (38.55%)
	Do not want to disclose	5 (0.57%)
Age	Male	Mean 23.80 ± 7.51
	Female	Mean 23.90 ± 6.41
Education Attainment	No elementary school	2 (0.23%), Mean age 18.50 ± 0.71
	Elementary school	181 (0.82%), Mean age 18.60 ± 1.39
	High school	539 (62.02%), Mean age 22.50 ± 5.30
	Baccalaureate degree	66 (7.60%), Mean age 28.9 ± 6.93
	University degree	70 (8.10%), Mean age 34.71 ± 9.89
	Postgraduate college	70 (8.10%), Mean age 34.71 ± 9.89
	PhD	4 (0.46%), Mean age 40.5 ± 15.61
Marital Status	Not involved in relationship	461 (53.04%), Mean age 21.02 ± 4.50
	In relationship	346 (39.81%), Mean age 22.2 ± 4.9
	Married	58 (6.70%), Mean age 39.1 ± 10.63
	Divorced	3 (0.34), Mean age 33.3 ± 13.5
	Widow	1 (0.11)
Type of Sport	Swimming	183 (21.05%)
	Soccer	173 (19.90%)
	Athletics	98 (11.27%)
	Tennis	86 (9.89%)
	Volleyball	53 (6.09%)
	Handball	33 (3.80%)
	Basketball	25 (2.87%)
	Boxing	22 (2.53%)
	Archery	16 (1.84%)
	Gymnastic	16 (1.84%)
Sport-Related Injury (Last 12 Months)	Yes No	245 (71.80%), Mean age 22.9 ± 7.30 624 (71.80%), Mean age 22.8 ± 7.02
Participation in Competitions (Last 12 Months)		Mean value 14.27 ± 12.01
Next Competition (Days)		Mean value 19.88 ± 32.80

Percentages are calculated from the total number of participants. Mean values are provided with standard deviations. Age data are reported separately for male and female athletes where applicable. Educational attainment categories reflect the highest level of education completed.

**Table 3:** Descriptive statistics for APSQ items.

	APSQ1	APSQ2	APSQ3	APSQ4	APSQ5	APSQ6	APSQ7	APSQ8	APSQ9	APSQ10
N	869	869	869	869	869	869	869	869	869	869
Median	1.00	2.00	2.00	2.00	2.00	3.00	2.00	2.00	1.00	1.00
Mean	1.65	1.81	1.88	1.79	1.87	2.50	2.48	2.17	1.19	1.22
SD	0.83	0.94	0.96	0.92	1.02	1.16	1.28	1.18	0.60	0.66
Min	1.00	1.00	1.00	1.00	1.00	1.00	1.00	1.00	1.00	1.00
Max	5.00	5.00	5.00	5.00	5.00	5.00	5.00	5.00	5.00	5.00

APSQ: Athlete Psychological Strain Questionnaire. The statistics include responses from 869 participants for each APSQ item. Median and mean values indicate the central tendency of responses for each item. Standard deviation provides information on the variability of responses. Minimum and maximum values indicate the range of responses for each item, with 1 being “Never” and 5 being “Always”. N—number of participants, SD—standard deviation, Min—minimum value, Max—maximum value.

**Table 4:** APSQ factor loadings and reliability statistics.

Variables	F1 Loading	F2 Loading	If-Item-Dropped Cronbach’s α	Item–Rest Correlation	Mean	SD
APSQ1 (It was difficult to be around teammates)	0.56	0.11	0.71	0.41	1.65	0.83
APSQ2 (I found it difficult to do what I needed to do)	0.80	−0.04	0.69	0.56	1.81	0.94
APSQ3 (I was less motivated)	0.79	−0.06	0.69	0.55	1.88	0.96
APSQ4 (I was irritable, angry, or aggressive)	0.78	−0.13	0.69	0.50	1.79	0.92
APSQ5 (I could not stop worrying about injury or my performance)	0.62	0.20	0.69	0.50	1.87	1.02
APSQ6 (I found training more stressful)	0.04	0.91	0.73	0.27	2.50	1.16
APSQ7 (I found it hard to cope with selection pressures)	−0.08	0.92	0.76	0.14	2.48	1.28
APSQ8 (I worried about life after sport)	0.58	0.23	0.70	0.48	2.17	1.18
APSQ9 (I needed alcohol or other substances to relax)	0.54	−0.06	0.72	0.35	1.19	0.60
APSQ10 (It was difficult to be around teammates)	0.53	−0.05	0.72	0.34	1.22	0.66
Explained Variance (Expl. Var.)	3.50	1.83				
Proportion of Total Variance (Prp. Totl.)	0.35	0.18				

APSQ: Athlete Psychological Strain Questionnaire. Factor loadings indicate the correlation between each variable and the identified factors. F1 and F2 refer to Factor 1 and Factor 2, respectively. Explained Variance (Expl. Var.) refers to the amount of variance in the data explained by each factor. Proportion of Total Variance (Prp. Totl.) indicates the proportion of total variance explained by each factor. The table shows also the reliability statistics for each item in the APSQ questionnaire. “If-item-dropped Cronbach’s α” indicates the Cronbach’s alpha value if that item is removed. “Item–rest correlation” shows the correlation between the item and the sum of the other items. “Mean” and “SD” represent the mean and standard deviation of the responses for each item, respectively.

## Data Availability

Data are available on reasonable request.
